# Himalayan uplift shaped biomes in Miocene temperate Asia: evidence from leguminous *Caragana*

**DOI:** 10.1038/srep36528

**Published:** 2016-11-09

**Authors:** Ming-Li Zhang, Xiao-Guo Xiang, Juan-Juan Xue, Stewart C. Sanderson, Peter W. Fritsch

**Affiliations:** 1 CAS Key Laboratory of Biogeography and Bioresource in Arid Land, Xinjiang Institute of Ecology and Geography, Chinese Academy of Sciences, Urumqi 830011, China; 2 State Key Laboratory of Systematic and Evolutionary Botany, Institute of Botany, Chinese Academy of Sciences, Beijing 100093, China; 3 Shrub Sciences Laboratory, Intermountain Research Station, Forest Service, U.S. Department of Agriculture, Utah 84601, USA; 4 Botanical Research Institute of Texas, Fort Worth, Texas 76107-3400, USA

## Abstract

*Caragana*, with distinctive variation in leaf and rachis characters, exhibits three centers of geographic distribution, i.e., Central Asia, the Qinghai-Tibetan Plateau (QTP), and East Asia, corresponding to distinct biomes. Because *Caragana* species are often ecologically dominant components of the vegetation in these regions, it is regarded as a key taxon for the study of floristic evolution in the dry regions of temperate Asia. Based on an expanded data set of taxa and gene regions from those previously generated, we employed molecular clock and biogeographical analyses to infer the evolutionary history of *Caragana* and link it to floristic patterns, paleovegetation, and paleoclimate. Results indicate that *Caragana* is of arid origin from the Junggar steppe. Diversification of crown group *Caragana*, dated to the early Miocene ca. 18 Ma and onwards, can be linked to the Himalayan Motion stage of QTP uplift. Diversification of the major clades in the genus corresponding to taxonomic sections and morphological variation is inferred to have been driven by the uplift, as well as Asian interior aridification and East Asian monsoon formation, in the middle to late Miocene ca. 12~6 Ma. These findings demonstrate a synchronous evolution among floristics, vegetation and climate change in arid Central Asia, cold arid alpine QTP, and mesophytic East Asia.

*Caragana* (Fabaceae), belonging to the legume tribe Hedysareae^1^, contains ca. 100 species distributed in temperate Asia, with most species occurring in China^2^,^3^ (Fig. 1). This genus exhibits distinctive morphological variation involving leaflet arrangement, either pinnate or palmate, and the leaf rachis, either deciduous and non-spiny, or persistent and spiny^2^,^4^. The geographic distribution of *Caragana* is thought to be fundamentally related to the divisions of vegetation, climate, and natural physical geography in China^2^,^3^,^5^, namely, the palmate leaf/persistent rachis group in arid steppe and desert, commonly in northwestern China; the pinnate leaf/persistent rachis group in the cold and arid alpine regions of the Qinghai Tibetan Plateau (QTP); and the pinnate leaf-deciduous rachis group in the temperate forests of East Asia and especially northern China. In these biomes, many *Caragana* species form dominant components of the natural vegetation^2^,^5^ (Fig. 1). Therefore, *Caragana* is regarded as a key taxon in understanding the floristics, vegetation, ecology, and climate of the dry regions of temperate Asia^3^,^5^,^6^.

Two hypotheses for the origin of *Caragana* have been proposed on the basis of morphological and cytological data, i.e., southern Balkhash Lake of the Central Asian Turan Basin^4^, or in the mesophytic forests of East Asia^6^,^7^. More recent studies based on molecular evidence^2^,^3^ support the recognition of three main clades (sections) within *Caragana*^2^ and suggest that the Himalayan Motion and rapid uplift of the QTP drove generic evolution, with the Junggar Basin proposed as an arid ancestral area. However, insufficient species sampling and the lack of the incorporation of parameters on current and past vegetation and climate have limited both phylogenetic and biogeographical inferences based on these studies. Here we increase both taxon sampling and sequence data relative to those used in previous studies to explore the evolutionary history of *Caragana* and to infer the degree of concordance of this history with the paleovegetation and paleoclimate of temperate Asia. We also assess the possible abiotic factors driving the diversification of the genus.

## Results

### Phylogenetic analysis and divergence time estimation

Maximum parsimony (MP), maximum likelihood (ML) and Bayesian inference (BI) were employed in phylogenetic analysis, and yielded topologically identical trees (Figs 2 and S3). The relaxed clock Bayesian trees (Figs 2 and S4) also yielded a comparable topology. Six clades correspond to the sections recognized in studies of morphology (Table S2), which are mainly characterized by the variation of leaflet type and leaf rachis persistence. The stem age of *Caragana* was estimated at ca. 29 Ma with normal priors (ca. 32 Ma with uniform priors), whereas the crown age was estimated at ca. 18 Ma with normal priors (ca. 20 Ma with uniform priors). Estimated crown ages of the six clades/sections range from ca. (13)12 to 6 Ma (Figs 2, 3 and S4). Dating with normal and uniform priors produced approximately estimated ages at some nodes especially at six nodes/sections, consistent with the crown ages recovered from previous studies, i.e., 29 Ma and 33 Ma^2^,^3^ of the legume tribes Hedysareae and Hedysareae + *Astragalus*, respectively (Fig. 2).

### Ancestral area reconstruction

Ancestral area analyses recovered an origin for crown-node *Caragana* as the Junggar and QTP by S-DIVA, Junggar and East Asia by DEC, and Junggar by BBM (Figs 3 and S5). The most likely state of the node is recovered as Junggar (D) by S-DIVA and DEC. Most other nodes also recovered D as the preponderant state, whereas steppe is preponderant as the ancestral biome (Figs 3 and S5). Sections *Bracteolatae* and *Jubata*e, with restricted distributions in the alpine QTP, are shown to be derived from early dispersals from Junggar at ca. 18 to 14 Ma (Fig. 2), followed by dispersal from the QTP to East Asia ca. 14 to 10 Ma. The analysis allowed inference of steppe as the original biome in the genus, followed by consecutive dispersals to forest, subalpine, and then alpine biomes. Section *Caragana* originated from the Junggar and dispersed to East Asia during ca. 12~5.8 Ma, in the sequence of steppe to forest.

### Diversification analysis

Sliding window analysis yielded accelerated diversifications in *Caragana* during ca. 20~16 Ma, 15~12 Ma, and 12~8 Ma, with the greatest development of taxa mainly at ca. 12~8 Ma (Fig. 3). BAMM analyses yielded a speciation rate that was initially high and then declined, and a positive extinction rate that increased slightly throughout the history of *Caragana* (Fig. 4). The net diversification rate was higher than the extinction rate prior to 5 Ma, though no support for heterogeneous diversification dynamics was found in this lineage (posterior probability of a single rate model = 1). In considering the influence of topology for the BAMM analyses, the mean value of probability shift over 100 trees was <0.05, which indicates that the results of 100 random trees are consistent with that of the MCC tree. The results of both sliding window and BAMM analyses show that *Caragana* developed most lineages prior to 5 Ma.

## Discussion

### The origin of *Caragana*

Based on sediments, crust, vegetation, palynology, and macrofossils of the Loess Plateau in eastern QTP etc., three phases of the QTP uplift have been proposed^8^. The first phase (Gangdese Motion 45–38 Ma) is characterized by the collision of the Indian and Asian plates, resulting in the rise of the Gangdese Mountains. The second phase (Himalayan Motion 25–17 Ma) encompasses the rise of the QTP to ca. 2,000 m or more, the westward withdrawal of the Paratethys Sea, the aridification of the Asian interior, and the onset of the Asian monsoon. The third phase involved the intense uplift of the QTP ca. 3.6 Ma, accompanied by the formation of the modern Asian monsoon. A rapid uplift has been suggested at 8 Ma^9^, although this is disputed^8^. Our date of ca. 18 Ma for the origin of crown-node *Caragana* falls within the Himalayan Motion of the QTP uplift stage, which can thus be presumed to have driven initial generic diversification. Paleoclimatic and paleovegetational evidence^8^,^9^,^10^,^11^,^12^,^13^,^14^,^15^,^16^ indicates that a broad arid zone with arid subtropical vegetation occurred across Eurasia prior to ca. 30 Ma until ca. 20 Ma (Oligocene to early Miocene), which broadly overlaps the current distribution of *Caragana*. QTP uplift altered the climate of Asia by obstructing the warm-humid airflow of the Indian Ocean and changed Asian climate and environmental pattern; the eastern part of this zone was subsequently pushed to northwestern China, and was replaced by a subtropical and humid zone in North China and the Hengduan Mountains of the eastern QTP, and by a cold arid zone in the QTP^8^,^13^. Because climate in the Junggar and northwestern China remained arid and the vegetation was unaltered as steppe over this period^11^,^12^,^13^,^14^,^15^,^16^,^17^,^18^, we infer a xeric and steppe origin for *Caragana* by inferring a Junggar ancestral area and biome (Fig. 3). This is in agreement with the arid hypothesis for the origin of the genus^2^,^3^,^4^ rather than an origin from mesophytic forest^7^.

### Diversification within *Caragana*

**Figure 1:**
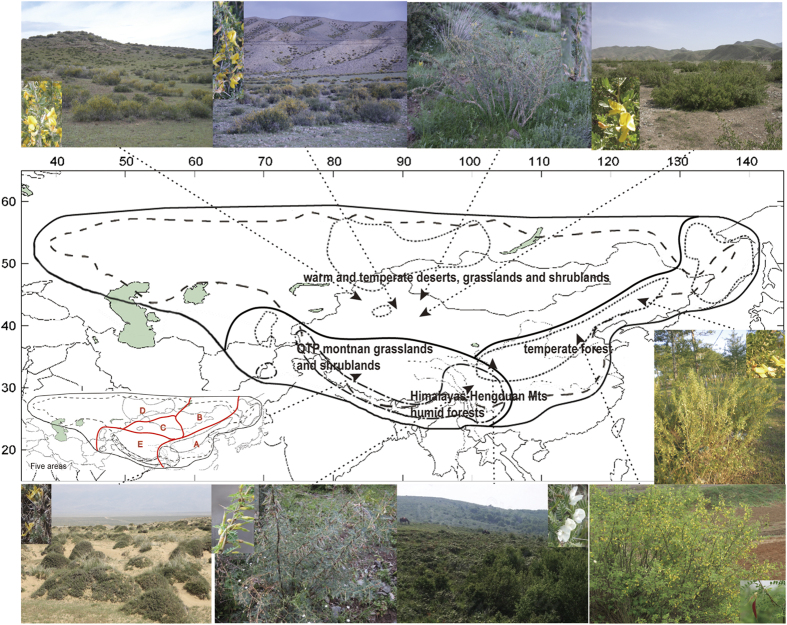
Distribution and vegetation of *Caragana*. Map^3^ (Springer journal/Plant Systematics and Evolution, Evolutionary response of *Caragana* (Fabaceae) to Qinghai–Tibetan Plateau uplift and Asian interior aridification, 288 (2010), 191–199, Zhang, M. L. & Fritsch, P. W., with permission of Springer) showing distribution of the genus *Caragana* and sections *Caragana* (dotted line), *Bracteolatae* (dash-dotted line), and *Frutescentes* (broken line). Central Asia, QTP and East Asia are indicated with thick line, and an inset depicting the division of five areas, are shown. Representatives of eleven species showing leaf rachis type and biome are, from left to right above map, *C. frutex* (steppe), *C. leucophloea* (desert), *C. tragacanthoides* (shrubland), *C. pleiophylla* (desert), and from left to right below map, *C. tibetica* [Qinghai-Tibetan Plateau (QTP)], *C. bicolor* (eastern QTP forest), *C. jubata* (alpine meadow), *C. purdomii* (forest), and nested in the map, *C. microphylla* (forest).

**Figure 2:**
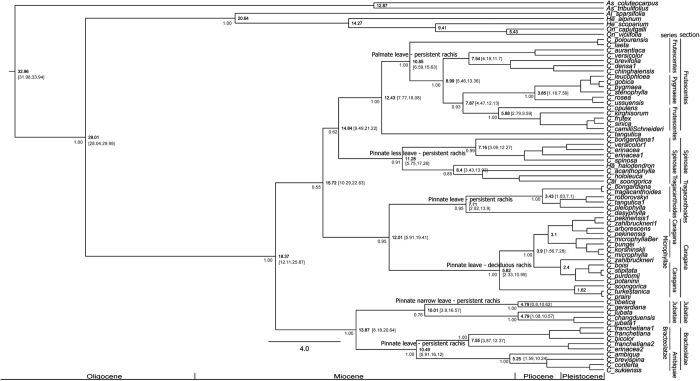
Phylogenetic tree. Phylogenetic relationships of *Caragana* and relatives based on a relaxed clock analysis (normal distribution priors) with seven gene regions. The sections within the genus, along with the characters of rachis persistence and leaflet arrangement, and 95% HPD and 95% confidence intervals, are shown.

**Figure 3:**
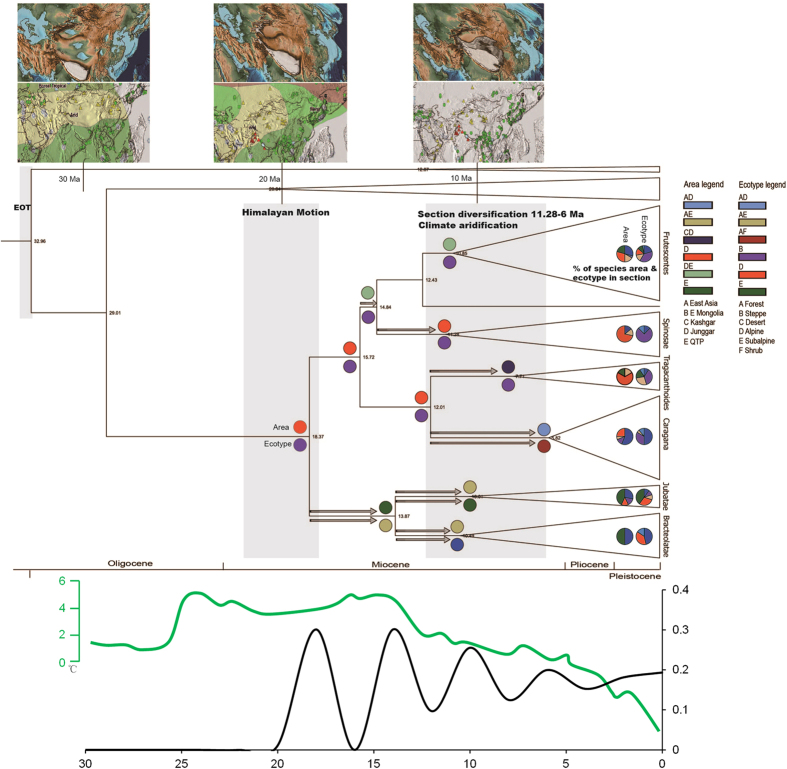
Ancestral area reconstruction and biome evolution. S-DIVA results for ancestral area reconstruction (AAR). Colored circles indicate the most likely state at nodes above branches for AAR, and below branches for biome evolution. Dispersals are shown by arrows. Maps of ancient geology, vegetation, and climate at three time slices, i.e., 30 Ma, 20 Ma, and 10 Ma (paleogeographic and paleoclimatic maps by C. R. Scotese with permission), are illustrated above the chronogram. Himalayan Motion, and Central Asian aridification are labelled. Global temperature curve estimate (thin green line)^21^, and *Caragana* diversification peaks calculated from Sliding Windows Analysis (thick black line) are indicated below the chronogram.

The diversification of *Caragana* in the three regions of Central Asia, QTP and East Asia corresponds well with the six taxonomic sections (clades) of the genus based on leaflet and rachis characters. Diversifications at the six nodes corresponding to these sections occurred ca. 126 Ma (Fig. 2), and also approximately at the ranges of the four peaks of 18(12)–8 Ma within *Caragana* as shown by sliding window analysis (Fig. 3). Specifically, sections *Bracteolatae* and *Jubata*e, with pinnate (or narrow) leaves and a persistent rachis, have generally restricted distributions in the alpine QTP. The species of the mainly East Asian section *Caragana*, characterized by pinnate leaves with a greater number of leaflets and a deciduous rachis, likely originated from the Junggar and dispersed to East Asia, in the sequence of steppe to forest, as interpreted from our analyses. Having originated in the ancestral biome of Junggar steppe, the arid Central Asian *Frutescentes, Spinosae*, and *Tragacanthoides* clades with the palmate leaves and a persistent rachis (Fig. 2), underwent dispersals in the QTP, Kashgar, Mongolia, and Turan, and diversified and adapted into steppe, desert or/and forest, alpine, subalpine and shrub biomes (Fig. 3). Two dispersals occurred from the Junggar, one to the QTP within section *Frutescentes*, the other to the Kashgar within section *Tragacanthoides*, and two dispersals occurred within the steppe biome (Figs 1 and 3). On the whole, the distinct morphological traits of the sections/clades in three regions not only demonstrate diversification within *Caragana* (Fig. 2), but also demarcate representative groups of three floras, vegetation types, biomes, and climate zones (Fig. 1). *Caragana* displays differentiation in each of these regions, i.e. the arid steppe and desert zone in northwestern China, the cold arid high-altitude zone in the QTP, and the temperate forest zone in East Asia^5^,^8^,^13^,^19^.

### *Caragana* evolution and abiotic dynamics

We can link the inferred evolutionary history and divergence time estimates in *Caragana* from our data to three aspects of geology and climate. Firstly, global cooling and aridification at the Eocene-Oligocene Transition (EOT) 33 Ma^17^,^20^,^21^, and Tethys retreat westward from early Oligocene 34~32 Ma^22^ and its closing at 21.5 Ma^11^, most likely triggered the arid origin of *Caragana* at about 29 Ma, which is estimated as the divergence (stem) time of the genus, and also the age of origin for the crown node of tribe Hedysareae^23^ (including *Caragana*). Both the EOT and Tethys retreat appear to have been the abiotic drivers of the arid origin of *Caragana* in the QTP and Central Asian regions^10^,^17^.

Second, a marked stage of QTP uplift, the Himalayan Motion, occurred at the southern QTP 25~17 Ma^8^, which profoundly affected biomes, vegetation and climatic patterns in Asia at 20~10 Ma^16^. The previous arid zone across Eurasia prior to ca. 20 Ma became restricted westward to Central Asia (including Junggar and northwestern China), whereas the humid zone was expanded in East Asia^8^,^12^,^13^,^16^. Accordingly, three zones of climate, vegetation and biome zones were formed in China, namely, the eastern humid, northwestern arid, and QTP high-cold zones. These zones correspond to the major patterns of morphological differentiation in *Caragana*. The crown-node age of *Caragana* of ca. 18 Ma falls within the range of the period of the Himalayan Motion. *Caragana* origin in the Junggar ancestral arid steppe and biome and later diversification can be considered as the long-term effects of arid climate driven by the Himalayan Motion.

**Figure 4:**
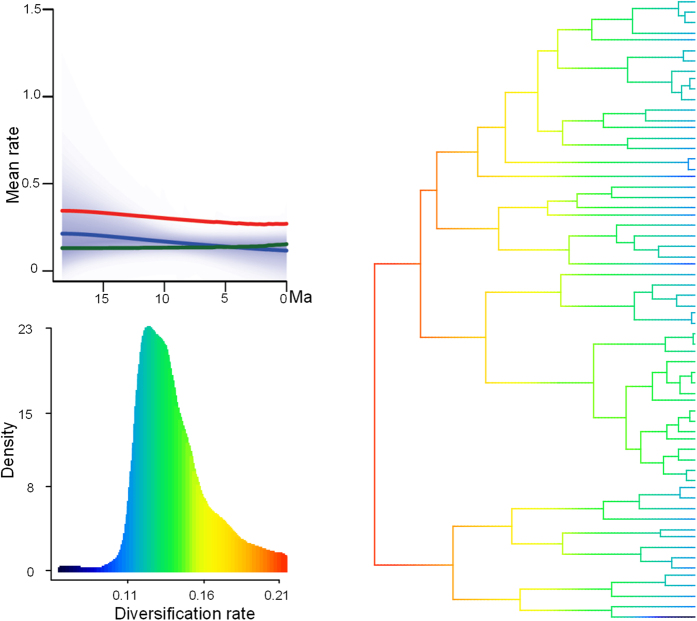
Diversification rates through time. The phylogeny shows a time-calibrated BEAST tree, with branches colored by reconstructed net diversification rates. The red line denotes the mean speciation rate, the blue line represents the mean net diversification, and the green line represents the mean extinction rate across *Caragana* through time.

Third, the lasting climatic aridification and other events following the Himalayan Motion during (13)12~6 Ma (Figs 2, 3, 4 and S4) possibly played a direct role in the diversification of the six *Caragana* clades/sections, and its divergence in the three regions. This is observed for the mesophytic group, mainly section *Caragana* in North China and the Hengduan Mountains, sections *Bracteolatae* and *Jubatae* in the QTP^2^,^3^, and the xerophytic sections *Frutescentes, Spinosae* and *Tragacanthoides* mainly in arid and semiarid northwestern China and Central Asia (Fig. 1). The abiotic events during (13)12~6 Ma responsible for the divergence of these group are likely the principal QTP uplift in 13~7 Ma^9^,^11^,^16^, CO_2_ concentration deceasing at ca. 12 Ma^24^ or 14~10 Ma^25^, Eurasian moisture and temperature change, especially Central Asian aridification 17~5 Ma^15^, global cooling and aridification at 8~7 Ma^26^, East Asian monsoon onset 9~8 Ma^27^, and higher accumulations of dust in the Loess Plateau in the eastern QTP 15~13 Ma and 8~7 Ma^13^. Of these, the QTP uplift and climate aridification are likely the most fundamental factors driving macroevolutionary abiotic dynamics. The comprehensive effect of these factors has likely drove diversification in *Caragana* and other plant lineages especially in the QTP and adjacent regions^18^,^28^,^29^. In summary, *Caragana* appears to well exemplify the evolutionary history of the flora and vegetation of temperate Asia, whereas its evolutionary history is illuminated by the abiotic events of QTP uplift, paleovegetation and paleoclimate.

## Methods

### Taxon sampling and DNA sequencing

Seventy-one species and samples were obtained from *Caragana* and outgroup species in the tribe Hedysareae and *Astragalus* (Supplementary Information Table S1). Seven DNA fragments, i.e., *nr*DNA ITS and *cp*DNA *rbc*L, *trn*G-S, *atp*B-*rbc*L, *psb*A-*trn*H, *psb*B-H, and *mat*K, were sequenced as described previously^2^,^30^. An aligned data set of 7601 nucleotide positions was used for phylogenetic dating. A partitioning strategy to test for phylogenetic conflict among the seven genes used Bayesian inference was conducted as in Xiang *et al*.^31^, the results of which showed that our data could be combined for phylogenetic analysis.

### Phylogenetic analysis and divergence time estimation

Phylogenetic analysis was conducted with MP, ML and BI analyses as previously described^2^. Bayesian phylogenetic analysis and divergence time estimates were simultaneously generated with a relaxed clock method in BEAST 1.5.4 (http://beast.bio.ed.ac.uk/). We used the uncorrelated lognormal relaxed clock model with a Yule process for the speciation model and GTR + I + G for the substitution model (estimated for the data set).

There are no fossils for *Caragana*, tribe Hedysareae, or for *Astragalus*, but there are rich fossils in other lineages of the Fabaceae and thus a family dating scheme is available^23^. Based on this scheme, we used a date of 33 Ma for the tree root prior in our analysis, and 29 Ma for the origin of the tribe Hedysareae. Priors were treated as normally distributed with a standard deviation of 0.5. Because these constraints are secondary calibrations, a uniform prior in BEAST was employed to reduce potential error^32^. All of our sampled taxa are included the IRLC (inverted repeat-lacking), which has an estimated age 39 Ma^23^. Thus, the uniform root prior was set to an upper bound of 39 Ma and a lower bound of 33 Ma, and that for tribe Hedysareae to an upper bound of 39 Ma and lower bound of 29 Ma.

A Markov chain Monte Carlo (MCMC) was run for a billon generations and sampled every 1,000 generations for the 7-gene data set. The stationarity of runs was examined with the effective sample size of each parameter >200. TreeAnnotator v 1.7.2 was used to summarize the post burn-in (10%) trees and their parameters. FigTree 1.3.1 was used to visualize the final tree.

### Biogeographical analysis

Ancestral area reconstruction (AAR) was conducted with Statistical Dispersal-Vicariance (S-DIVA) analysis, likelihood analysis under the dispersal-extinction-cladogenesis (DEC) model, and Bayesian binary MCMC (BBM). Analyses were implemented in RASP v 3.2 [http://mnh.scu.edu.cn/soft/blog/RASP/]. Ancestral area and biome reconstructions (Table S1) were analyzed separately. Based on floristics^11^,^12^,^33^, vegetation^5^,^11^,^12^, and climate^11^,^12^,^13^, we divided the distribution of *Caragana* into five areas: East Asia (A), eastern Mongolia (B), Kashgar (C), Junggar (D), and QTP (E). These divisions comprise two larger categories, East Asia (A, E) and Central Asia (B–D). The former comprises Far East-northeastern China, northern China, the Hengduan Mountains, and the eastern Himalayas, all with humid forest vegetation, as well as the QTP. The latter comprises eastern Mongolia with semi-humid steppe, Kashgar (mainly western Mongolia and the Tarim Basin) with arid desert, and the Junggar, i.e., the Junggar and Turan basins, with arid steppe and desert. *Caragana* species can occur in various vegetation zones, such as alpine meadow, shrub, forest, steppe, or desert, and most are endemic and dominant in their communities (Fig. 1). Biomes were therefore divided into six types: forest, steppe, desert, alpine meadow, sub-alpine meadow, and shrub (Table S1).

The BEAST molecular dating tree (Fig. 2) was treated as a fully resolved phylogram and 1000 post-burn in trees derived from BEAST in RASP. RASP was performed with constraints of maximum areas 2 at each node, to infer possible ancestral areas and biomes, as well as potential vicariance and dispersal events. The outgroups used in the phylogeny were also used in the biogeographical analyses throughout data processing, but are not shown in the results.

### Diversification analysis

We used a sliding window analysis to visually examine the diversification rate change over time^34^. The analysis was carried out with the BEAST MCC tree and with the time span from present to 29 Ma divided into two-million-year windows. For each window, the number of new taxa that originated during the sliding window was divided by the number of taxa present prior to the start of the respective sliding window.

We also used Bayesian analysis of macro-evolutionary mixtures (BAMM)^35^ to infer speciation rates across the phylogeny of *Caragana*. The analyses were run on randomly sampled 100 BEAST MCC trees. Chains were run BAMM for 10 million generations and sampled every 10000 generations. The first 10% as burn-in and the effective sample size for likelihood and number of shifts was calculated to assess convergence. Event data generated from BAMM was analyzed with the R package BAMMtools^36^ to estimate rate-through-time dynamics and the number of evolutionary regime shifts from the posterior sampling. We used “global Sampling Fraction = 0.8” to set the sampling probability. Also, we completed 100 trees sampled from BEAST trees as above.

## Additional Information

**How to cite this article:** Zhang, M.-L. *et al*. Himalayan uplift shaped biomes in Miocene temperate Asia: evidence from leguminous *Caragana*. *Sci. Rep*. **6**, 36528; doi: 10.1038/srep36528 (2016).

**Publisher’s note**: Springer Nature remains neutral with regard to jurisdictional claims in published maps and institutional affiliations.

## Supplementary Material

Supplementary Information
