# Supplementary material for: Himalayan uplift shaped biomes in Miocene temperate Asia: evidence from leguminous Caragana
Source: Sci Rep. 2016 Nov 9;6:36528. doi: 10.1038/srep36528 (PMC5101512; doi:10.1038/srep36528)

Himalayan uplift shaped biomes in Miocene temperate Asia: evidence from leguminous *Caragana*

Ming-Li Zhang, Xiao-Guo Xiang, Juan-Juan Xue, Stewart C. Sanderson & Peter W. Fritsch

Table S1

Seven-gene sequence data used for reconstructing the phylogenetic tree and divergence time dating, and species area and biome data used for biogeographical analysis.

| Taxon | Voucher | Source | GenBank accession number (ITS, *rbc*L, *trn*S-G*, atp*B-*rbc*L, *psb*A-*trn*H, *psb*B-H, *mat*K) | Distribu  -tion area | Biome |
| --- | --- | --- | --- | --- | --- |
| **Sect. *Caragana*** |  |  |  |  |  |
| **Ser. *Caragana*** |  |  |  |  |  |
| *C. arborescens* Lam. | *M.L. Zhang 00-201* (PE) | Altai, Xinjiang, China | FJ537262, FJ537211, FJ537164, ----------  ----------, ----------, ---------- | AD | A |
| *C. boisii* C. K. Schneid. | *M.L. Zhang & Y. Kang 00-121* (PE) | Lixian, Sichuan, China | FJ537259, FJ537208, FJ537161, ----------  ----------, ----------, ---------- | A | A |
| *C. potaninii* Kom. | *Z.Y. Chang ?* (WUG) | Wutai, Shanxi, China | -----------, -----------, -----------, ----------  ----------, ----------, ---------- |  | A |
| *C. prainii* C. K. Schneid. | *D. Podlech 16678* (MSB) | Kunar, Afghanistan | FJ537255, FJ537205, FJ537157, ---------  ----------, ----------, ---------- | D | B |
| *C. purdomii* Rehder | *C.Y. Chang et al. 2004059* (WUG) | Yan’an, Shaanxi, China | FJ537261, FJ537710, FJ537163, ----------  ----------, ----------, ---------- | A | A |
| *C. soongorica* Grubov | *M.L. Zhang* 00-256 (PE) | Cultivated, Urumqi Botanical Garden, Xinjiang, China | FJ537257, FJ537207, FJ537159, ----------  ----------, ----------, ---------- | D | BF |
| *C. stipitata* Kom. | *Y. Kang 00-55* (PE) | Huashan (Qingling), Shaanxi, China | FJ537260, FJ537209, FJ537162, ----------  ----------, ----------, ---------- | A | A |
| *C. turkestanica* Kom. | *M.L. Zhang 00-101*(PE) | Cultivated, Bergius Botanical Garden, Stockholm, Sweden | FJ537256, FJ537206, FJ537158, ----------  ----------, ----------, ---------- | D | AF |
| *C. zahlbruckneri* C. K. Schneid. | *S.Y. He 18765* (PE) | Zhangjiakou, Hebei, China | FJ537258, ----------, FJ537160, ----------  ----------, ----------, ---------- | A | A |
| *C. zahlbruckneri* C. K. Schneid. 1 | *Z.Y. Chang ?* (WUG) | ? Hebei, China | -----------, -----------, -----------, -----------  ----------, ----------, ---------- |  |  |
| **Ser. *Microphyllae* (Kom.) Pojark.** |  |  |  |  |  |
| *C. bungei* Ledeb. | *M.L. Zhang et al. 99-225* (PE) | Bajanchongor, Mongolia | FJ537267, FJ537216, FJ537169, ----------  ----------, ----------, ---------- | B | B |
| *C. korshinskii* Kom. | *M.L. Zhang 00-149* (PE) | Cultivated, TBG, Xinjiang, China | FJ537266, FJ537215, FJ537168, ----------  ----------, ----------, ---------- | C | BC |
| *C. microphylla* Lam.1 | *M.L. Zhang et al. 99-214* (PE) | Lhongcheng, Mongolia | FJ537264, FJ537213, FJ537166, ----------  ----------, ----------, ---------- | AB | AB |
| *C. microphylla* Lam. 2 | *M.L. Zhang 177-99-74-80* (PE) | Cultivated, Berlin Botanical Garden, Germany; originally from northern China | FJ537265, FJ537214, FJ537167, ----------  ----------, ----------, ---------- |  |  |
| *C. pekinensis* Kom. | *M.L. Zhang 99-56* (PE) | Xiangshan, Beijing, China | FJ537263, FJ537212, FJ537165, ----------  ----------, ----------, ---------- | A | A |
| *C. pekinensis* Kom. 1 | *M.L. Zhang* *177-99-74-80?* (PE) | Cultivated, Berlin Botanical Garden, Germany; originally from Beijing, China | -----------, -----------, -----------, -----------  ----------, ----------, ---------- | A | B |
| **Sect. *Bracteolatae* (Kom.) M. L. Zhang** |  |  |  |  |  |
| **Ser. *Bracteolatae* Kom.** |  |  |  |  |  |
| *C. bicolor* Kom. | *M.L. Zhang & Y. Kang Y 99-178* (PE) | Markang, Sichuan, China | FJ537246, FJ537197, FJ537147, ----------  ----------, ----------, ---------- | A | ADF |
| *C. brevispina* Benth. | *M.L. Zhang 281-05-8414/101* (PE) | Cultivated, Berlin Botanical Garden, Germany (originally from Kashmir) | FJ537248, FJ537200, FJ537150, ----------  ----------, ----------, ---------- | E | AD |
| *C. franchetiana* Kom. | *M.L. Zhang & S. Z. Zhang 94-178* (WUG) | Gongbujiangda, Xizang, China | –, FJ537198, FJ537148, ----------  ----------, ----------, ---------- | A | ADF |
| *C. franchetiana* Kom.1 | *M.L. Zhang* ? (KUN) | Zhongdian, Yunnan, China | -----------, -----------, -----------, -----------  ----------, ----------, ---------- |  |  |
| *C. franchetiana* Kom.2 | *L.R. Xu 0081* (WUG) | Xiangcheng, Sichuan, China | -----------, -----------, -----------, -----------  ----------, ----------, ---------- |  |  |
| *C. sukiensis* C. K. Schneid. | *S.G. Miehe & K. Kock s.n.* (NHM) | Donkardzong, Nepal | FJ537247, FJ537199, FJ537149, ----------  ----------, ----------, ---------- | A | AD |
|  |  |  |  |  |  |
| **Ser. *Ambiguae* Sanchir** |  |  |  |  |  |
| *C. ambigua* Stocks | *R.P. Steward 28001* (KEW) | Baluchistan, Pakistan | -----------, -----------, -----------, -----------  ----------, ----------, ---------- |  |  |
| *C. conferta* Benth. ex Baker | *J.F. Duthie 12192* (NHM) | Astor-Gudhui, Kashmir | FJ537250, –, FJ537152, ---------- |  |  |
|  |  |  |  |  |  |
| **Sect. *Jubatae* (Kom.) Y. Z. Zhao** |  |  |  |  |  |
| **Ser. *Jubatae* Kom.** |  |  |  |  |  |
| *C. jubata* (Pall.) Poir. | *M.L. Zhang 00279* (PE) | Zhaosu (Tianshan), Xinjiang, China | FJ537242, FJ537194, FJ537143, ----------  ----------, ----------, ---------- | ADE | ADE |
| *C. pleiophylla* (Regel) Pojark. | *M.L. Zhang 10-146* (PE) | Tekes, Xinjiang, China | FJ537253, FJ537203, FJ537155, ----------  ----------, ----------, ---------- | D | BCE |
| *C. roborovskyi* Kom. | *M.L. Zhang 00-88* (PE) | Uhai, Nei Mongol, China | FJ537254, FJ537204, FJ537156, ----------  ----------, ----------, ---------- | D | C |
| *C. tangutica* Maxim. | *Q.L. Ho et al. 2499* (NHM) | Yushu, Qinghai, China | FJ537278, FJ537227, FJ537180, ----------  ----------, ----------, ---------- | E | AEF |
| *C. tangutica* Maxim.1 | *M.L. Zhang 07-02* (PE) | Jiayuguan, Gansu, China | -----------, -----------, -----------, -----------  ----------, ----------, ---------- | E | AEF |
| **Ser. *Leucospinae* Y. Z. Zhao** |  |  |  |  |  |
| *C. changduensis* Y. X. Liou | *Z.C. Ni et al. 1069* (PE) | Chayü, Xizang, China | FJ537243, –, FJ537144, ----------  ----------, ----------, ---------- | A | DE |
| *C. gerardiana* Benth. | *S.G. Miehe & K. Kock K 01-032-03* (NHM) | Western Nepal | FJ537245, FJ537196, FJ537146, ---------- | AE | DE |
| *C. tibetica* (Maxim. ex C. K. Schneid.) Kom. | *M.L. Zhang 00-89* (PE) | Uhai, Nei Mongol, China | FJ537244, FJ537195, FJ537145, ----------  ----------, ----------, ---------- | CD | BCE |
|  |  |  |  |  |  |
| **Sect. *Frutescentes* (Kom.) Sanchir** |  |  |  |  |  |
| **Ser. *Frutescentes* Kom.** |  |  |  |  |  |
| *C. camilli-schneideri* Kom. | *C.Y. Chang et al. 2004334* (WUG) | Yumin, Xinjiang, China | FJ537283, FJ537232, FJ537184, ----------  ----------, ----------, ---------- | D | B |
| *C. frutex* (L.) K. Koch | *M.L. Zhang 177-97-74-80* (PE) | Cultivated, BLBG, Germany | FJ537285, FJ537234, FJ537186, ----------  ----------, ----------, ---------- | D | AB |
| *C. kirghisorum* Pojark. | *C.Y. Chang et al. 2004219* (WUG) | Khorgos, Xinjiang, China | FJ537280, FJ537229, FJ537181, ----------  ----------, ----------, ---------- | D | B |
| *C. laeta* Kom. | *M.L. Zhang 177-98-74-80* (PE) | Cultivated, BLBG, Germany | FJ537281, FJ537230, FJ537182, ----------  ----------, ----------, ---------- | D | B |
| *C. opulens* Kom. | *M.L. Zhang & Y. Kang 99-123* (PE) | Daofu, Sichuan, China | FJ537282, FJ537231, FJ537183, ----------  ----------, ----------, ---------- | AE | BDE |
| *C. polourensis* Franch. | *B. Bartholomew et al. 9417* (CAS) | Minfeng (Kunlun), Xinjiang, China | FJ537279, FJ537228, –, ----------  ----------, ----------, ---------- | C | C |
|  |  |  |  |  |  |
| **Ser. *Chamlagu* Pojark.** |  |  |  |  |  |
| *C. rosea* Turcz. ex Maxim. | *M.L. Zhang 99-45* (PE) | Beihuashan, Beijing, China | FJ537272, FJ537221, FJ537174, ----------  ----------, ----------, ---------- | A | A |
| *C. sinica* (Buc’hoz) Rehder | *M.L. Zhang 99-49* (PE) | Xiangshan, Beijing, China | FJ537284, FJ537233, FJ537185, ----------  ----------, ----------, ---------- | A | A |
| *C. ussuriensis* (Regel) Pojark. | *M.L. Zhang* *00-113* (PE) | Cultivated, UPBG, Sweden | FJ537273, FJ537222, FJ537175, ----------  ----------, ----------, ---------- | A | A |
|  |  |  |  |  |  |
| **Ser. *Pygmaeae* Kom.** |  |  |  |  |  |
| *C. aurantiaca* Koehne | *M.L. Zhang 00-156* (PE) | Cultivated, TBG, Xinjiang, China | FJ537270, FJ537219, FJ537172, ----------  ----------, ----------, ---------- | ADE | BDE |
| *C. brevifolia* Kom. | *Q.L. Ho et al. 2498* (NHM) | Yushu, Qinghai, China | FJ537268, FJ537217, FJ537170, ----------  ----------, ----------, ---------- | AE | BE |
| *C. chinghaiensis* Y.X. Liou | *Q.L. Ho et al.* 93 (CAS) | Tongde, Qinghai, China | FJ537269, FJ537218, FJ537171, ----------  ----------, ----------, ---------- | E | AD |
| *C. gobica* Sanchir | *M.L. Zhang et al. 99-304* (PE) | Gobi-Altai, Mongolia | FJ537277, FJ537226, FJ537179, ----------  ----------, ----------, ---------- | C | BC |
| *C. leucophloea* Pojark. | *M.L. Zhang et al. 99-218* (PE) | Daxinchileng, Mongolia | FJ537275, FJ537224, FJ537177, ----------  ----------, ----------, ---------- | CD | BC |
| *C. pygmaea* (L.) DC. | *M.L. Zhang 00-187* (PE) | Jinghe, Xinjiang, China | FJ537276, FJ537225, FJ537178, ----------  ----------, ----------, ---------- | CD | BC |
| *C. stenophylla* Pojark. | *M.L. Zhang* 00-78 (PE) | Hangjinqi, Nei Mongol, China | FJ537274, FJ537223, FJ537176, ----------  ----------, ----------, ---------- | B | B |
| *C. versicolor* Benth. | *S. Miehe 99-62-06* (NHM) | Upper Dolpo, Nepal | FJ537271, FJ537220, FJ537173, ----------  ----------, ----------, ---------- | E | D |
| *C. versicolor* Benth. 1 | *Z.Y. Chang ?* (WUG) | ? Xizang, China | -----------, -----------, -----------, -----------  ----------, ----------, ---------- | E | D |
| *C. densa* Kom. 1 | *L.R. Xu ?* (MSB) | Hongyuan,Sichuan,China | -----------, -----------, -----------, -----------  ----------, ----------, ---------- | A | AEF |
|  |  |  |  |  |  |
| **Sect. *Spinosae* (Kom.) Y. Z. Zhao** |  |  |  |  |  |
| **Ser. *Spinosae* Kom.** |  |  |  |  |  |
| *C. bongardiana* (Fisch. & C. A. Mey.) Pojark. | *M.L. Zhang 00215* (PE) | Jimunai, Xinjiang, China | FJ537251, FJ537201, FJ537153, ----------  ----------, ----------, ---------- | D | B |
| *C. bongardiana* (Fisch. & C. A. Mey.) Pojark. 1 | *Z.Y. Chang ?* (WUG) | ? Xinjiang, China | -----------, -----------, -----------, -----------  ----------, ----------, ---------- | D | B |
| *C. erinacea* Kom. 1 | *Z.Y. Chang ?* (WUG) | Hongyuan,Sichuan,China | -----------, -----------, -----------, -----------  ----------, ----------, ---------- | A | AF |
| *C. hololeuca* Bunge ex Kom. | *M.L. Zhang 00-153* (PE) | Cultivated, TBG, Xinjiang, China | FJ537240, FJ537192, FJ537141, ----------  ----------, ----------, ---------- | D | B |
| *C. spinosa* (L.) Hornem. | *C.Y. Chang et al. 2004503* (WUG) | Qinghe, Xinjiang, China | FJ537241, FJ537193, FJ537142, ---------- | C | B |
| *C. tragacanthoides* (Pall.) Poir. | *C.Y. Chang et al. 2004404* (WUG) | Hebukesaier, Xinjiang, China | FJ537252, FJ537202, FJ537154, ----------  ----------, ----------, ---------- | D | B |
|  |  |  |  |  |  |
| **Ser. *Acanthophyllae* Pojark.** |  |  |  |  |  |
| *C. acanthophylla* Kom. | *M.L. Zhang 00-154* (PE) | Cultivated, TBG, Xinjiang, China | FJ537238, FJ537191, FJ537139, ----------  ----------, ----------, ---------- | D | B |
|  |  |  |  |  |  |
| **Ser. *Dasyphyllae* Pojark.** |  |  |  |  |  |
| *C. dasyphylla* Pojark. | *Xinjiang Expedition Team 472* (WUG) | Kuche, Xinjiang, China | FJ537239, –, FJ537140, ----------  ----------, ----------, ---------- |  |  |
| *C. dasyphylla* Pojark. 1 | *Z.Y. Chang ?* (WUG) | ? Xinjiang, China | -----------, -----------, -----------, -----------  ----------, ----------, ---------- |  |  |
|  |  |  |  |  |  |
| **Outgroups** |  |  |  |  |  |
| *Calophaca soongorica* Kar. & Kir. | *E.E. Pyoahobeq & L.A. Kpamapehko 5-14-1984* (PE) | Semiipalatinskaya, Tajikstan | FJ537288, FJ537237, FJ537189, ----------  ----------, ----------, ---------- | D | B |
| *Calophaca soongorica* Kar. & Kir. 1 | *Z.Y. Chang ?* (WUG) | ? Xinjiang, China | -----------, -----------, -----------, -----------  ----------, ----------, ---------- |  |  |
| *Halimodendron halodendron* (Pall.) Voss. | *M.L. Zhang 00-279* (PE) | Cultivated, URBG, Xinjiang, China | FJ537289, FJ537237, FJ537190, ----------  ----------, ----------, ---------- | D | B |
| *Hedysarum alpinum* L. | *M. Riewe 182* (CAS) | Northwest Territories, Canada | FJ537287, FJ537235, FJ537188, ----------  ----------, ----------, ---------- |  |  |
| *Hedysarum scoparium* Fisch. et Mey | *H.H. Meng* | Cultivated, TBG, Xinjiang, China | -----------, -----------, -----------, -----------  ----------, ----------, ---------- |  |  |
| *Onobrychis caput-galli* (L.) Lam. | ?(PE) | Bulgaria | -----------, -----------, -----------, -----------  ----------, ----------, ---------- |  |  |
| *Onbrychis viciifolia* Scop | ?(PE) | Poland | -----------, -----------, -----------, -----------  ----------, ----------, ---------- |  |  |
| *Alhagi sparsifelia* Shap. | *H.H. Meng* | Cultivated, TBG, Xinjiang, China | -----------, -----------, -----------, -----------  ----------, ----------, ---------- |  |  |
| *Sphaerophysa* *salsula* (Pall.) DC. | *H.H. Meng* | Cultivated, TBG, Xinjiang, China | -----------, -----------, -----------, -----------  ----------, ----------, ---------- |  |  |
| *Astragalus coluteocarpus* Boiss. | *Qinghai-Xizang Expedition Team 76-8083* (PE) | Zada, Ali, Xizang, China | FJ537286, –, FJ537187, ----------  ----------, ----------, ---------- |  |  |

*Astragalus* *tribulifo-*

*lius* Benth. ex Bunge *H.N. Qin* *et al. 608* (PE), Lhasa, Xizang AF521953, -----------, -----------, -----------

----------, ----------, ----------

____________________________________________________________________________________________________________________________________________________________

^a^The classification of *Caragana* follows Zhang (1997).

Five distribution areas of *Caragana*, A: East Asia, B: eastern Mongolia, C: Kashgar, D: Junggar, and E: Tibet; six biomes, A: forest, B: steppe, C: desert, D: alpine, E: sub-alpine, and F: shrub.

BLBG: Berlin Botanical Garden; BGBG: Bergius Botanical Garden; TBG: Turfan Botanical Garden; UPBG: Uppsala Botanical Garden; URBG: Urumqi Botanical Garden.

Table S2. Sectional classification of *Caragana* used in the present study. A revised species classification based on phylogenetic analyses will be presented elsewhere.

| Section | *Caragana* |
| --- | --- |
| Section | *Bracteolatae* (Kom.) M. L. Zhang |
| Section | *Jubatae* (Kom.) Y. Z. Zhao |
| Section | *Tragacanthoides* (Pojark.) M. L. Zhang |
| Section | *Spinosae* (Kom.) Y. Z. Zhao |
| Section | *Frutescentes* (Kom.) Sanchir |

Figure S3. Phylogenetic tree as reconstructed by maximum parsimony (MP), maximum likelihood (ML), and Bayesian inference (BI). Numbers at nodes from left to right are MP, ML, and BI support values.


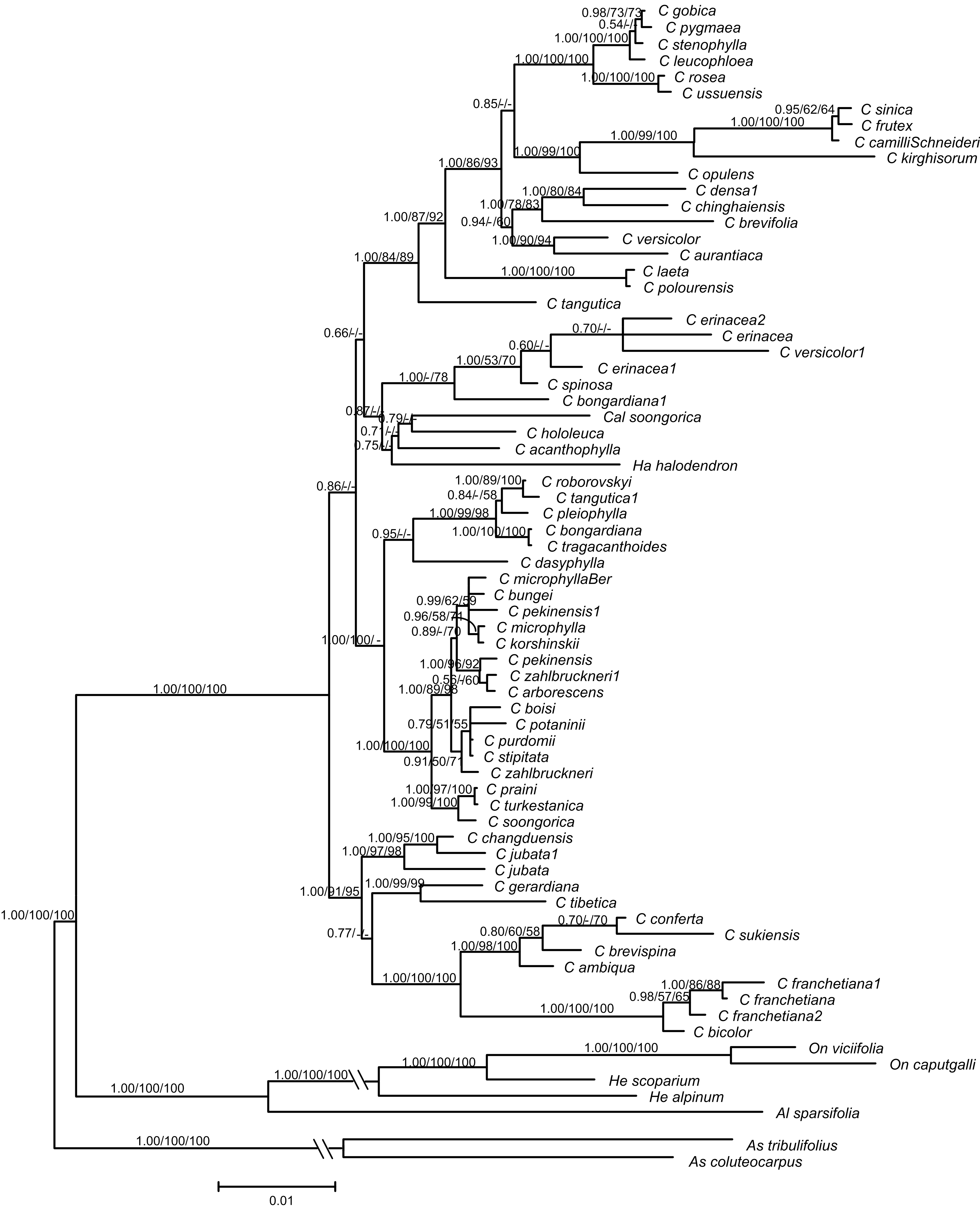


Figure S4. Phylogenetic relationships of *Caragana* and relatives based on a relaxed clock analysis (priors of uniform distribution) with seven gene regions. The 95% HPD and 95% confidence intervals are shown at six nodes and others (see Fig. 2).


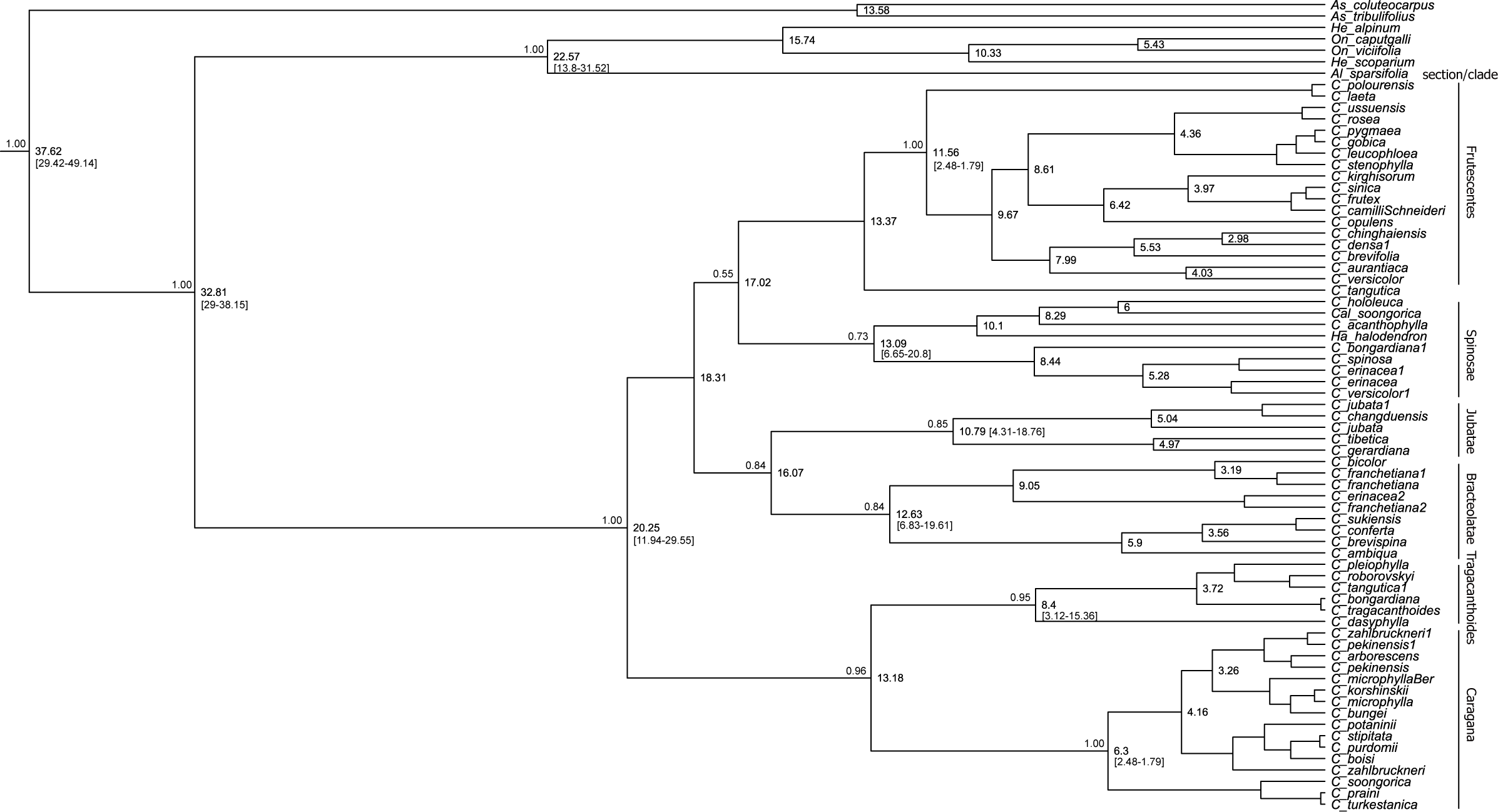


Figure S5. Ancestral area reconstruction (AAR) and biome evolution. Pie charts at nodes above branches refer to AAR, below branches to biome state. From left to right are those results from S-DIVA, DEC, and BBM analyses.


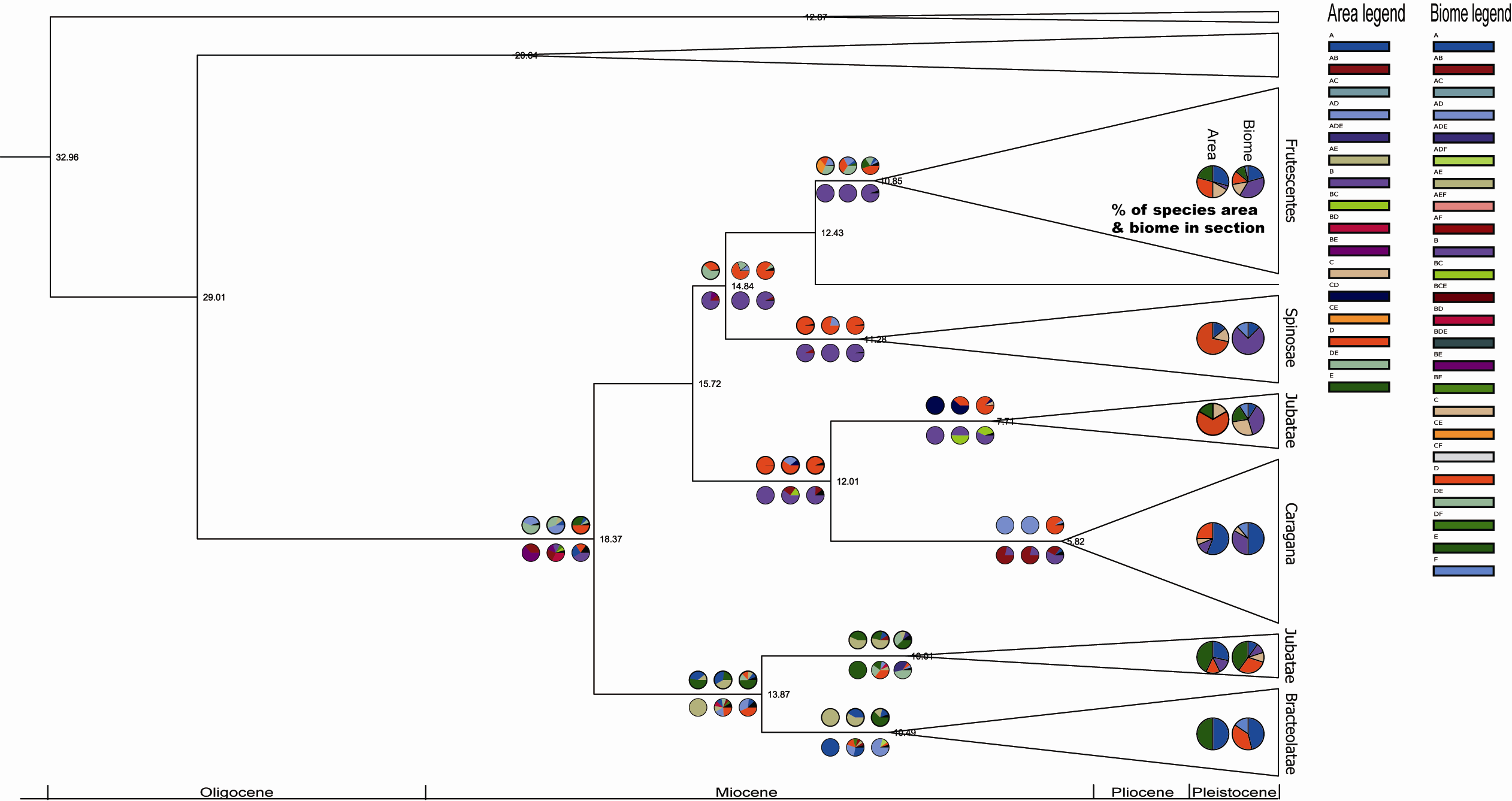

Supplement: Supplementary Information [file srep36528-s1.doc]
